# Influence of Strengthened Nodes on the Mechanical Performance of Aeolian Sand–Geogrid Interface

**DOI:** 10.3390/ma16134665

**Published:** 2023-06-28

**Authors:** Wei Du, Rusong Nie, Yongchang Tan, Jie Zhang, Yanlu Qi, Chunyan Zhao

**Affiliations:** 1 School of Civil Engineering, Central South University, Changsha 410075, China; 214812326@csu.edu.cn (W.D.); tangyc202306@163.com (Y.T.); zhangjie_dzgcx@chd.edu.cn (J.Z.); zcyzbb@163.com (C.Z.); 2 MOE Key Laboratory of Engineering Structures of Heavy Haul Railway, Central South University, Changsha 410075, China; 3 Xinjiang Railway Survey and Design Institute Co., Ltd., Urumqi City 830011, China; tyyqyl@163.com

**Keywords:** geogrid, aeolian sand, pull-out test, strengthened nodes, discrete element

## Abstract

Node thickening is a way to strengthen the nodes of a geogrid. Increasing the node thickness in conventional biaxial geogrids enhances the interface frictional strength parameters and improves its three-dimensional reinforcement effect. Based on the triaxial tests of aeolian sand, single-rib strip tests of geogrids, and pull-out tests of geogrid in aeolian sand, a three-dimensional discrete element pull-out model for geogrids with strengthened nodes was developed to investigate the mechanical performance of an aeolian sand–geogrid interface. The influences of increasing node thickness, the number of strengthened nodes, and the spacing between adjacent nodes on the mechanical performance of the geogrid–soil interface were extensively studied used the proposed model. The results demonstrated that strengthened nodes effectively optimize the reinforcing performance of the geogrid. Among the three node-thickening methods, that in which both the upper and lower sides of nodes are thickened showed the most significant improvement in ultimate pull-out resistance and interface friction angle. Moreover, when using the same node-thickening method, the ultimate pull-out resistance increase shows a linear relationship with the node thickness increase and the strengthened node quantity. In comparison with the conventional geogrid, the strengthened nodes in a geogrid lead to a wider shear band and a stronger ability to restrain soil displacement. When multiple strengthened nodes are simultaneously applied, there is a collective effect that is primarily influenced by the spacing between adjacent nodes. The results provide a valuable reference for optimizing the performance of geogrids and determining the spacing for geogrid installation.

## 1. Introduction

The utilization of geogrids in soil structure reinforcement has gained widespread acceptance in engineering practice, attributed to their notable economic advantages and exceptional mechanical performance [1,2,3,4]. The behavior and interaction at the interface between the soil and the geogrid directly influences the structural stability. The longitudinal and transverse ribs of the geogrid contribute to frictional resistance and passive resistance, effectively restraining soil movement. The pull-out test has been widely acknowledged by scholars [5,6] as a suitable method for studying the load transfer mechanism and pertinent parameters determining reinforced soil structures. Numerous studies have been conducted to explore the interaction characteristics between soil and geogrids. For instance, Lopes et al. [7] investigated the influence of confining pressure, fill density, and pulling velocity on the pull-out resistance of uniaxial geogrids. They observed that under consistent conditions, the pull-out resistance exhibited an increase with higher levels of confining pressure, fill density, or pull-out speed. Saleh et al. [8] performed pull-out tests on geogrids with different aperture sizes in combination with five different sub-ballast types characterized by distinct grading curves. Their findings indicated the presence of an optimum aperture size for a railway ballast with varying particle size distributions. Departing from this optimal size resulted in a diminished interlocking or reduced frictional force, consequently reducing the pull-out resistance. Cao et al. [9] compared the reinforcing performance of biaxial and triaxial geogrids through pull-out tests. They found that the strain distribution of biaxial geogrids was relatively uniform, while triaxial geogrids exhibited more concentrated strain near the pull-out end. Miao et al. [10] investigated the microscopic characteristics of various geogrids, including shear bands, force chains, microstructure, and the distribution of axial forces in the longitudinal ribs. Introducing the inscribed circle size–particle size ratio, they found that although the aperture geometry was different, triaxial geogrids and biaxial geogrids were homogenous in picking the optimal filler particle size. Wang et al. [11] investigated the influence of different loading modes on the microscopic characteristics. Their research emphasized notable disparities between the results obtained using rigid top loading and flexible top loading methodologies. 

As research has progressed, geosynthetic materials have evolved toward three-dimensional structural configurations. Zhang et al. [12] introduced the concept of three-dimensional reinforcement and investigated multiple strategies for its implementation. Through triaxial compression tests, they investigated the effect of different geogrid forms and spacings on the strength of sand. Li et al. [13] affixed geotextiles of different thicknesses to the nodes of a geogrid, creating a geogrid with three-dimensional reinforcement effects. They found that increasing node thickness shows a linear relationship with the ultimate pull-out resistance increase within a certain range. However, beyond that range, the relationship became inconclusive. Qiu et al. [14] applied the discrete element method to develop a two-dimensional numerical model simulating a reinforced embankment utilizing geogrids with strengthened nodes. They observed that the reinforced embankment exhibited reduced cumulative settlement and a more uniform distribution of internal stresses compared with unreinforced embankments. Zhang et al. [15] examined the impact of different methods for increasing node thickness on the results of pull-out tests. However, they did not provide a conclusive explanation for the mechanism underlying the influence of different methods for thickening the nodes. Beyranvand et al. [16] utilized concrete blocks attached to geogrids, resulting in a significant improvement in the passive resistance of the geogrids. Despite the valuable literature available, the mentioned studies have predominantly focused on conventional geogrids or have remained at the macroscopic level, leaving room for further investigation.

The microscopic mechanism underlying the enhanced reinforcement performance of geogrids with strengthened nodes remains unclear. Therefore, a numerical model was developed to investigate the mechanism through which different types of geogrids influence the results. The study confirmed the presence of a collective effect among nodes and identified the optimal spacing between adjacent nodes, offering references for the optimization design of geogrids with strengthened nodes. This study compared the shear band range of conventional geogrids and geogrids with strengthened nodes. It evaluated the three-dimensional reinforcement effect of geogrids with different thicknesses of strengthened nodes, providing valuable references for determining the optimal laying spacing.

## 2. Laboratory Tests and Numerical Models

### 2.1. Materials and Triaxial Test

**Figure 1:**
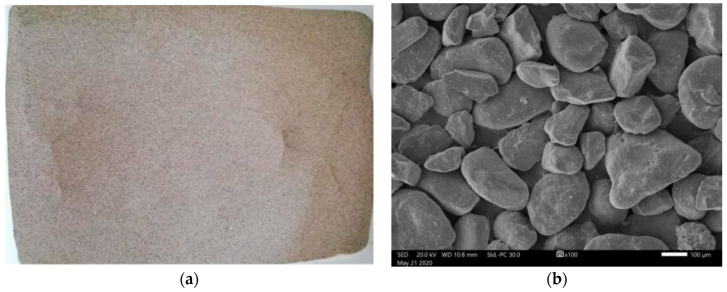
Photos of aeolian sand. (**a**) Digital photo. (**b**) SEM photo.

The test sample was aeolian sand obtained from the Taklamakan Desert. Figure 1 displays digital and scanning electron microscope (SEM) images of the aeolian sand. Despite the relatively uniform appearance of aeolian sand particles, they exhibit microscopic edges and corners that are not visible to the naked eye. 

The triaxial tests were conducted using a TSZ automatic triaxial apparatus, with samples having a diameter of 39.1 mm and a height of 80 mm. The sample preparation process followed the guidelines outlined in the Standard Test Methods for Soil Mechanics (T50123-2019) standard. Due to the low strength and limited cohesion of aeolian sand, it is recommended to avoid using filter paper at the bottom of the saturation tank and applying Vaseline around the sample. The test should be conducted immediately after specimen preparation to prevent potential issues arising from incomplete or rough surfaces caused by a large amount of sand sticking around the saturation chamber. The specimens were prepared with a compaction coefficient of K = 0.93 at the optimum water content. The consolidated-drained static triaxial compression test was conducted at a confining pressure of 30 kPa. Table 1 provides the basic physical property parameters of the aeolian sand [17].

**Figure 2:**
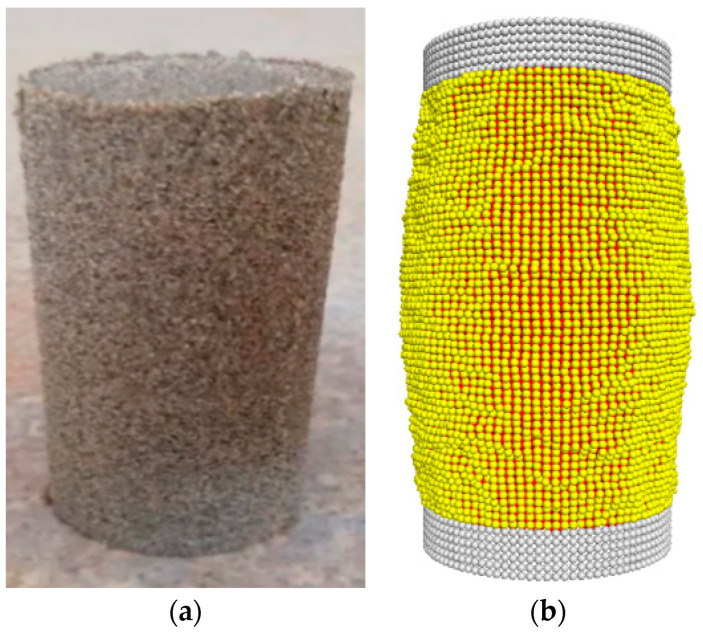
The triaxial test and particle flow simulation. (**a**) Physical sample for triaxial testing. (**b**) Numerical model for triaxial testing.

Figure 2 shows the numerical model of the triaxial test established using Particle Flow Code. The dimensions of the numerical triaxial model were matched to those of the physical model. The “distribute” command was utilized to simulate the aeolian sand particles. A rolling resistance linear model was applied for aeolian sand, considering the irregular shape of the actual particles. A sample containing 42,159 particles was selected to ensure a representative representation of the material characteristics. By applying prepressure to the test using a multisided wall, the larger internal stress within the sample was released. To generate flexible membrane particles based on the coordinates of surrounding walls, the walls were then removed, and confining pressure was applied by the flexible membrane particles. The contact between particles in the flexible membrane was simulated using a linear contact bond model, where the particle radius was set to 0.64 mm. Table 2 presents the microscopic parameters of the flexible membrane, mainly based on the tensile test of rubber membranes [18].

### 2.2. Validating the Numerical Model of the Triaxial Test

**Figure 3:**
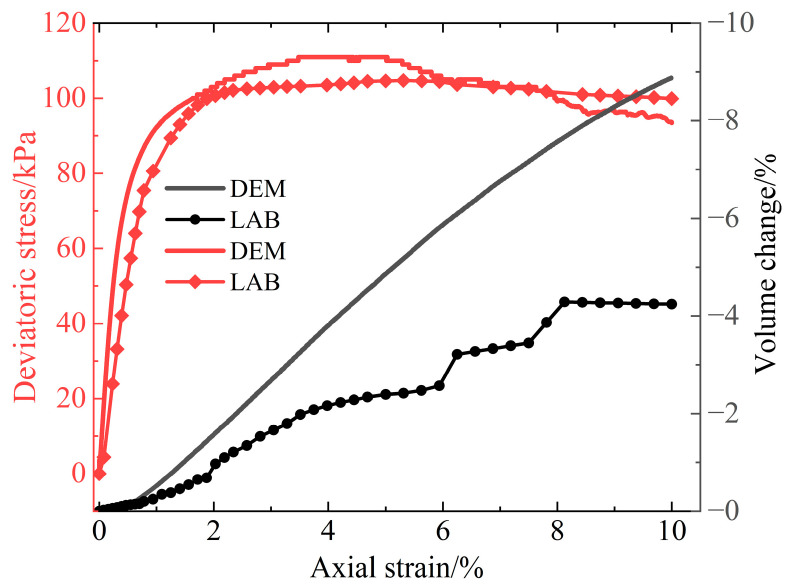
Deviatoric stress and volume strain–axial strain curves.

The contact between aeolian sand particles was simulated using the parameters provided in Table 3. Figure 3 shows the calibration results of the triaxial tests under a confining pressure of 30 kPa. The deviator stress–axial strain curve demonstrates a consistent overall development trend with minimal error. However, there is a significant disparity in volume variation between the numerical model and the physical model. This discrepancy primarily arises from two factors. Firstly, there are different particle sizes and shapes between the numerical model and the physical model. Secondly, the simulated particles lack the ability to fragment into smaller particles to match the soil’s pore spaces. Instead, they respond through misalignment and rearrangement under high-stress boundary conditions, thereby magnifying the effects of shear expansion within the simulation [19].

### 2.3. Tensile Test of Single Geogrid

**Figure 4:**
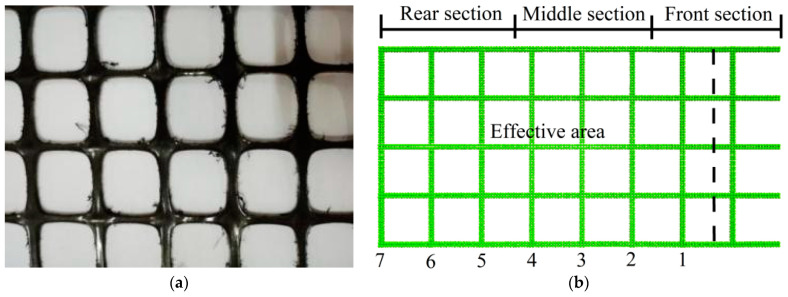
Detailed diagrams of geogrid. (**a**) Conventional biaxial geogrid. (**b**) Numerical model of the geogrid.

Figure 4a illustrates the biaxial geogrid used in this experiment, which was subjected to single geogrid tensile tests. The testing instrument was a ZNYT072 microcomputer-controlled electronic universal testing machine. The gauge length of the specimen on the instrument was set to 235 mm, with a tensile speed of 50 mm/min. For the experiment, five specimens with transverse ribs and five specimens with longitudinal ribs were prepared. 

Figure 2 shows the numerical model of the tensile test established using Particle Flow Code. The numerical simulation model of geogrid remained approximate to the actual size. The linear parallel bond model was used to simulate the contact between the geogrid particles. This model was resistant to tension but not compression, making it suitable for simulating the tensile properties of geogrids. We used the “fix” command to fix one end of the geogrid and applied a constant horizontal velocity to the other end.

### 2.4. Validating the Numerical Model of the Tensile Test

**Figure 5:**
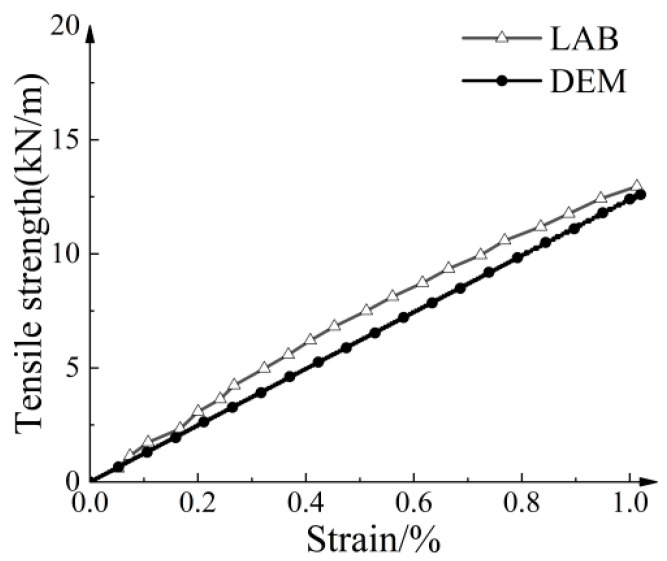
Comparison of stress–strain relationship of geogrid between tensile test and numerical model.

Table 4 displays the dimensions of the geogrid and the macromechanical parameters obtained from the tensile tests. Table 5 lists the microscopic parameters of the geogrid. Figure 5 compares the stress–strain relationship curve of the conventional geogrid. The simulated results demonstrated a strong agreement with the experimental results. 

## 3. Pull-Out Test and Numerical Model

### 3.1. Pull-Out Test

**Figure 6:**
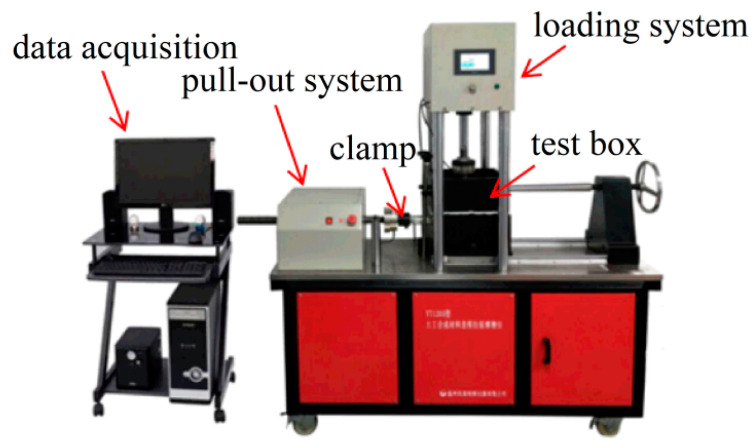
Pull-out test apparatus.

Figure 6 shows the YT1200S direct shear-pull friction apparatus, with test box dimensions of 250 × 200 × 200 mm. The soil sample was filled in four layers, each with a height of 50 mm. The geogrid used was a regular geogrid without reinforcement nodes, with a length of 280 mm. The embedded length in the soil was 250 mm, leaving 30 mm for connection with the fixtures. The width of the geogrid was 150 mm, maintaining a distance of 25 mm from the sides of the test box. After filling and leveling the first two layers of soil, the geogrid was installed. During installation, lubricant was applied to the pull-out port to prevent any frictional interference. Then, the soil was continuously layered up to the top of the test box, resulting in a total height of 200 mm. A rigid load plate was placed on the top surface of the soil sample, and normal pressure is applied, maintaining it for 15 min to ensure close contact between the aeolian sand and the geogrid. Then, the pull-out test was conducted. Considering the different burial depths of the geogrid in the roadbed, the normal stress for the pull-out test was determined as 15 kPa, 30 kPa, and 60 kPa, based on references from the relevant literature such as railway engineering [20]. The pull-out speed was set to 0.5 mm/min, and the test was terminated when the pull-out displacement reached 15 mm. Five parallel tests were conducted for each type of soil sample under the same normal stress to reduce the variability in the test results.

### 3.2. Numerical Model of Pull-Out Test

**Figure 7:**
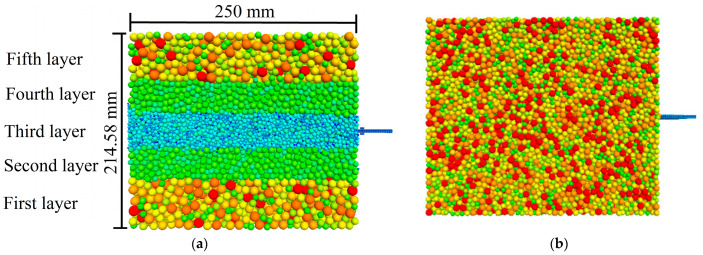
Numerical models of aeolian sand. (**a**) Layered aeolian sand numerical model. (**b**) Homogeneous aeolian sand numerical model.

**Figure 8:**
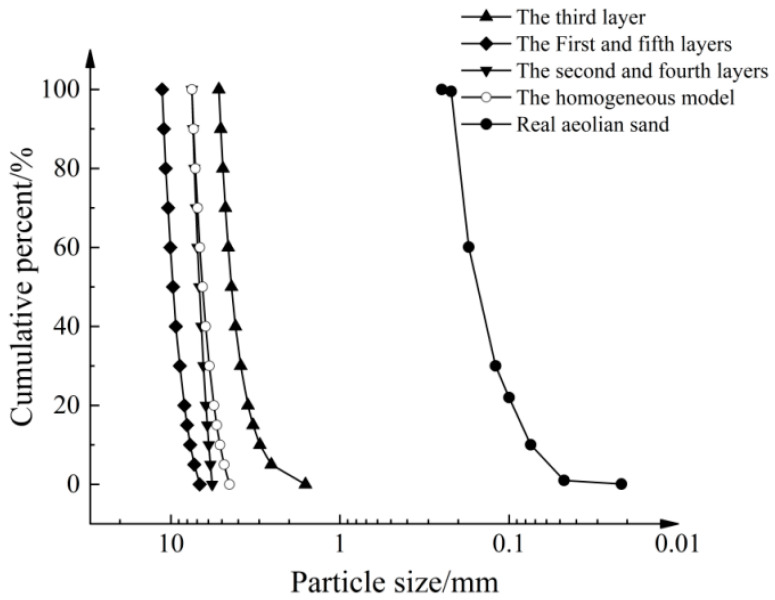
Gradation curve of simulated particles and aeolian sand.

The geometric dimensions of the numerical model for the pull-out test were aligned with those of the physical test. To improve computational efficiency, the size of the simulated particles can be scaled up by several tens of times or more. However, this approach inherently adversely affects the accuracy of the numerical model. Research has indicated that the use of excessively large magnification factors results in continuous rotation and sliding of the particles along the geogrid [21]. This phenomenon leads to denser particle arrangements and fluctuations in the pull-out force after reaching the peak. To investigate the influence of the magnification factor, a numerical model of layered aeolian sand was created. The magnification factor decreased as the distance from the geogrid decreased. In addition, a homogeneous numerical model of aeolian sand was generated to perform a comparative analysis. The soil sample was generated using the particle repulsion method. Figure 7a illustrates the numerical model of layered aeolian sand, consisting of 5 layers and a total of 64,425 particles. Figure 7b depicts a homogeneous numerical model with a particle size distribution ranging from 4.5 to 7.5 mm and a median particle size (D_50_) of 6.5 mm. The homogeneous model consisted of a total of 50,041 particles. The simulated particle and the grain size distribution curves of the actual aeolian sand are depicted in Figure 8. In the layered numerical model, the magnification factor of the sand particles near the interface is the smallest. The particle size distribution ranges from 1.6 to 5.2 mm, with a median particle size (D_50_) of 4.38 mm and a thickness of 35 mm. The second and fourth layers have a particle size distribution ranging from 5.7 to 7.5 mm, with a thickness of 35 mm for each layer. The first and fifth layers have a particle size distribution ranging from 6.75 to 11.25 mm, with an approximate thickness of 80 mm for each layer. A theoretical study showed that as long as a scale-dependent microscale constitutive model is selected, both the enlarged and original specimens exhibit the same mechanical response [22]. However, it is crucial to ensure that the number of particles in the 3D simulation model exceeds 40,000. Additionally, it is recommended to maintain a ratio between the external dimensions of the numerical sample and the average particle size that is less than 30 [23], taking into account the available computational capabilities for the simulation. Miao and Li utilized a pull-out speed of 0.375 m/s in their research to achieve quasistatic pull-out conditions [24,25]. In this study, a fixed horizontal velocity was applied to the pull-out end using the Fish function. Through several trials, it was observed that the force–displacement stabilizes when the pull-out speed is below 1 m/s. In this study, the pull-out velocity was set to 0.2 m/s, and the simulation was terminated when the pull-out displacement reached 15 mm. The changes in the pull-out force, particle displacement, porosity, and strain of the geogrid were monitored and recorded at regular intervals of 100 steps through the pull-out process.

### 3.3. Verification of the Numerical Model

**Figure 9:**
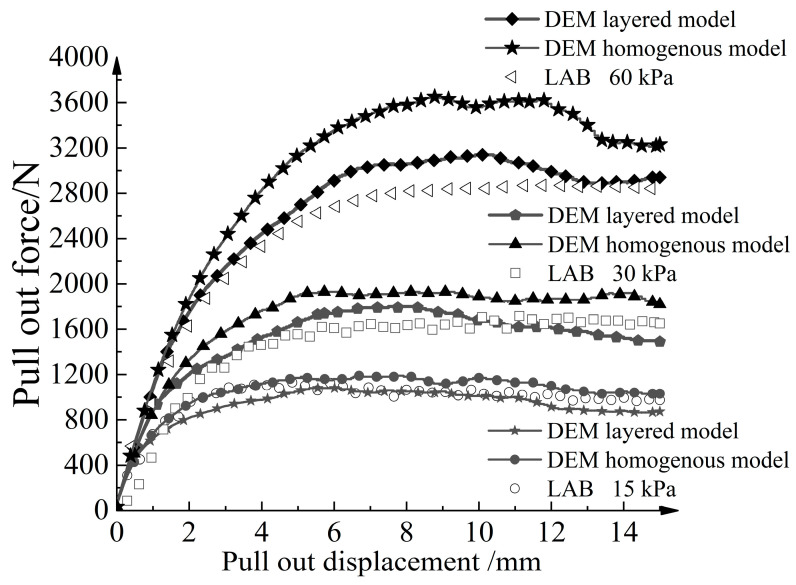
Relationship curve between pull-out force and pull-out displacement.

Figure 9 shows the pull-out results of the numerical models and the physical model. The initial stage demonstrates a linearly increasing trend in the curve, while the rate of increase in the pull-out force gradually decreases with the increase in pull-out displacement. After reaching the peak, the results of the numerical simulation exhibit a slight downward fluctuation in the curve. With an increase in normal stress, the curve shows an overall upward trend, and the pull-out displacement corresponding to the peak value increases. Under the same normal stress, the simulation curve closely matches the experimental curve, showing similar developmental trends. This confirms the rationality of both the layered numerical model and the homogeneous numerical model. When the normal stress is 15 kPa and 30 kPa, the overall pull-out force versus pull-out displacement curve of the homogeneous numerical model is slightly higher than that of the layered numerical model. This discrepancy arises from the presence of larger particles in the homogeneous aeolian sand model, leading to the geogrid being more prone to becoming stuck during the pulling process. Moreover, the stress concentration phenomenon near the rigid front wall becomes more pronounced.

### 3.4. Strengthening of Nodes and Design Conditions

**Figure 10:**
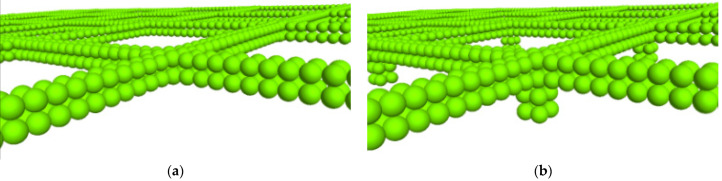

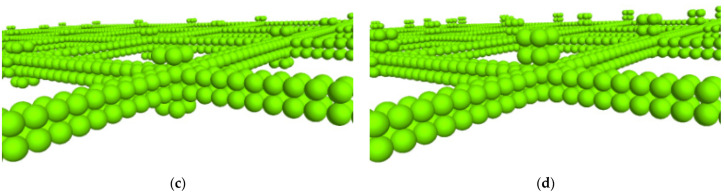
Different node-thickening methods. (**a**) Regular nodes (Condition A). (**b**) Strengthened nodes (Condition B). (**c**) Strengthened nodes (Condition C). (**d**) Strengthened nodes (Condition D).

As depicted in Figure 10, three types were investigated to examine the mechanical impact of different node thickening methods: strengthening on each individual side of the grid nodes and simultaneously strengthening on both sides. Condition A represented a standard biaxial geogrid without strengthened nodes. Condition B represented the thickening of the lower side of the nodes by 4.4 mm, while condition C represented the simultaneous thickening of both sides of the nodes by 2.2 mm each. Condition D included the thickening of the upper side of the nodes by 4.4 mm. To investigate the effect of the strengthened node thickness on the mechanical behavior of the geogrid–soil interface, Conditions E, F, and G were considered. In these conditions, the node thickness was increased by 4.4 mm, 6.6 mm, and 8.8 mm, respectively, applied to both the upper and lower sides of the grid nodes.

## 4. Analysis of Calculation Results

### 4.1. Influence of Node-Thickening Methods on Ultimate Pull-Out Resistance

**Figure 11:**
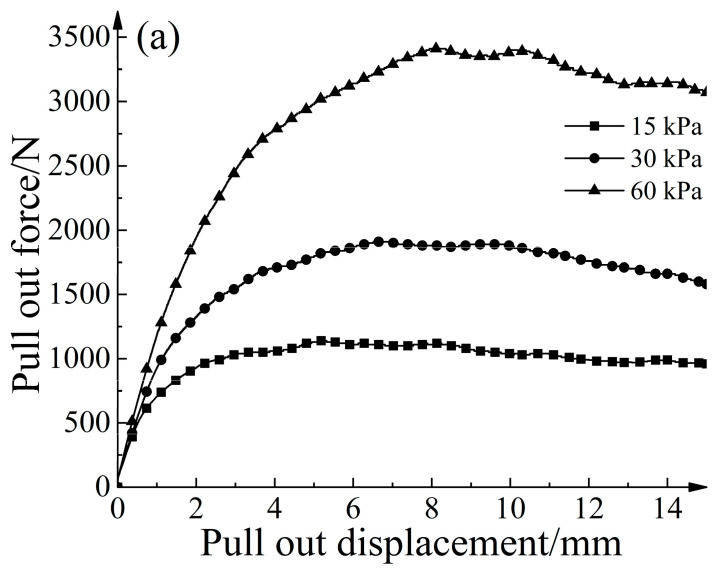

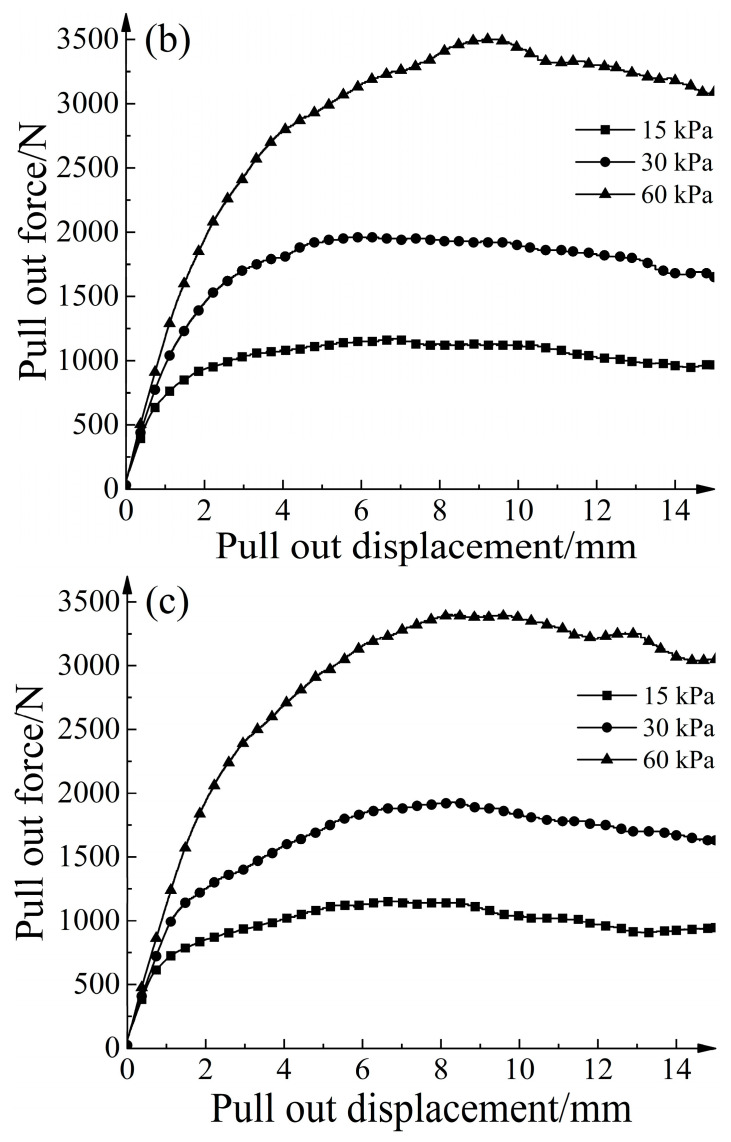
The relationship curve between pull-out force and pull-out displacement for different methods of increasing node thickness. (**a**) Pull-out force in Condition B. (**b**) Pull-out force in Condition C. (**c**) Pull-out force in Condition D.

Figure 11 depicts the relationship between pull-out resistance and displacement for different methods of increasing node thickness under various normal stresses. It is evident that strengthening the nodes significantly enhances the ultimate pull-out resistance. For instance, in the case of a normal stress of 30 kPa, the conventional geogrid demonstrated an ultimate pull-out resistance of 1800 N. In contrast, Condition B enhanced the ultimate pull-out resistance to 1910 N, representing a 6.11% improvement compared with the conventional geogrid. Condition D exhibited an ultimate pull-out resistance of 1930 N, which is a 7.22% improvement compared with the conventional geogrid. Condition C demonstrates an ultimate pull-out resistance of 1960 N, indicating an 8.89% improvement compared with the conventional geogrid. When the material used remained constant, Condition C exhibited the highest increase in ultimate pull-out resistance compared with Conditions B and D. Similar trends were observed under different normal stresses.

**Figure 12:**
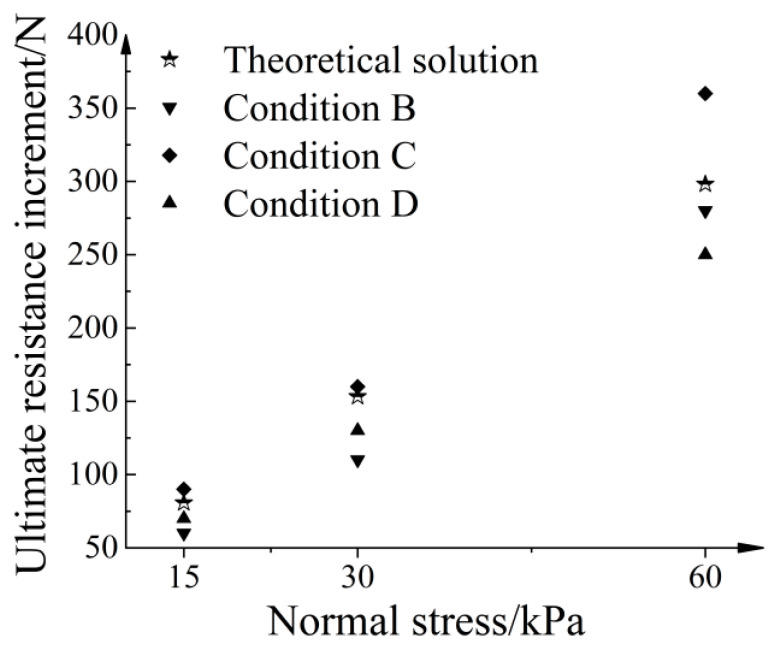
Comparison of numerical and theoretical solutions.

Jewell et al. [26] proposed the classic insertion shear theory, which was shown in previous research [27] to closely match the experimental values under low normal pressures. However, significant discrepancies emerge at higher normal stresses. The calculation method for the bearing stress $\sigma_{b}$ of the transverse ribs is as follows:

(1) $$\sigma_{b}=e^{(\pi /2+\varphi )\tan\varphi }\tan\left(\frac{\pi }{4}+\frac{\varphi }{2}\right)\sigma_{n}^{\prime }$$

where $\sigma_{n}^{\prime }$ represents the normal stress acting on the geogrid, including the constant normal stress applied by the loading plate and the normal stress generated by the overlying aeolian sand. φ is the internal friction angle of the aeolian sand (in radians). After calculating the specific values of $\sigma_{b}$, the increase in ultimate pull-out resistance provided by the strengthened nodes can be calculated using the following equation [10]:

(2) $$T=1.5NWH\sigma_{b}$$

where H is the thickness increase of the strengthened nodes, W is the width of the strengthened nodes, and N is the number of strengthened nodes. Figure 12 illustrates a comparison between the theoretical solution based on the punch-shear failure theory and the numerical solution. Overall, the numerical solutions for different conditions demonstrate reasonable agreement with the theoretical solutions, thereby validating the simulated node methods.

### 4.2. Influence of Node Thickening Methods on Geogrid–Soil Interface Strength Parameters

**Figure 13:**
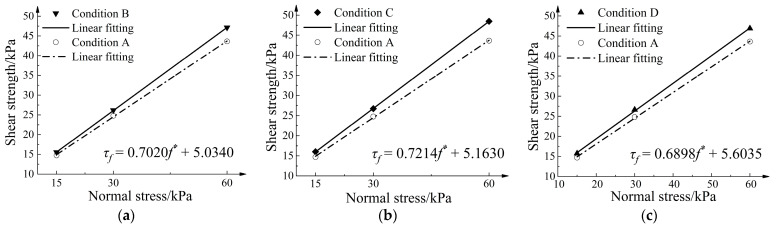
Shear strength–normal stress fitting curve. (**a**) Condition B; (**b**) Condition C; (**c**) Condition D, f* is the apparent friction coefficient.

Figure 13 presents the fitting curve for the shear strength–normal pressure relationship. Assuming static equilibrium and a uniform distribution of shear stress on the interface, the relationship between interface shear strength and normal stress is linearly fitted using the following equation, which enables the inverse calculation of the interface strength:

(3) $$\tau_{f}=\sigma_{n}f^{*}+C_{sg}$$

where $\sigma_{n}$ is the normal stress, $f^{*}$ is the apparent friction coefficient, and $C_{sg}$ is the apparent cohesion. Table 6 presents the interface frictional strength parameters for different node-thickening methods. As shown in the table, the variation in apparent cohesion is small, fluctuating between 5.1 kPa and 5.6 kPa. After node thickening, the interface friction angle increases by 2~3°. The most significant increase is observed when both sides of the nodes are simultaneously thickened.

### 4.3. Influence of Different Thicknesses of Strengthened Nodes on Ultimate Pull-Out Resistance

**Figure 14:**
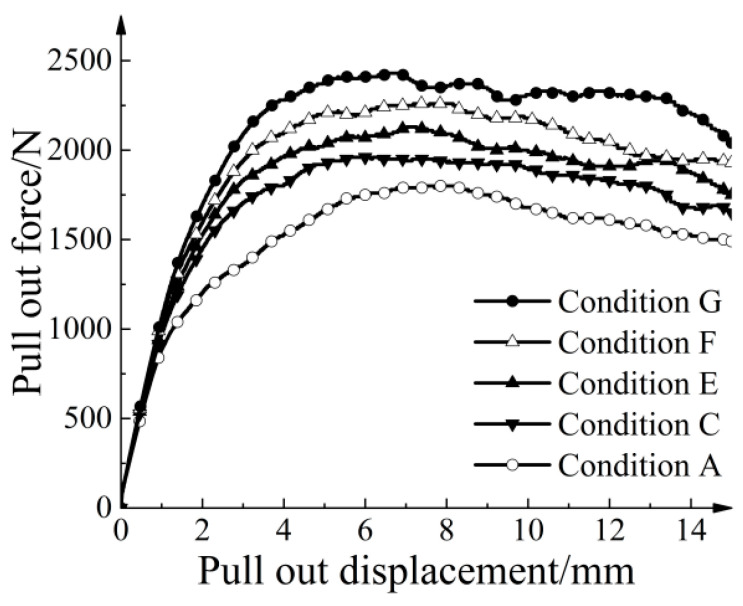
Pull-out force versus pull-out displacement.

Figure 14 shows the relationship between pull-out force and pull-out displacement for different thicknesses of strengthened nodes under 30 kPa. In Condition C, the ultimate pull-out resistance of the geogrid was 1960 N, which is 8.89% higher than that of the regular geogrid (condition A). In Condition G, the ultimate pull-out resistance of the geogrid was 2430 N, exhibiting a 35.00% increase in peak resistance. As the node thickness increased, the increase in ultimate pull-out resistance provided by the nodes also increased.

**Figure 15:**
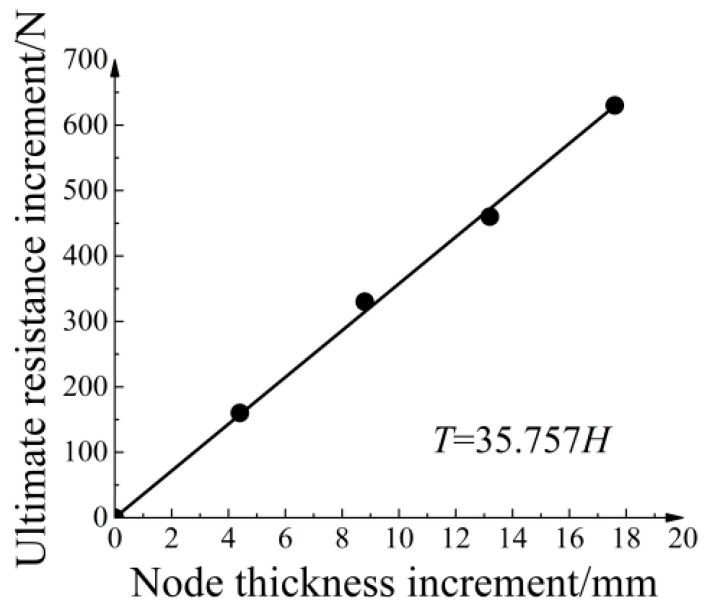
Relationship between node thickness increase and ultimate pull-out resistance increase.

In this study, the node thickness increase refers to the sum of the thickness increased on the upper and lower sides of the node. Figure 15 shows a clear linear relationship between the node thickness increase and the ultimate pull-out resistance increase. The fitted equation is expressed as T=35.757H, where *H* represents the thickness of node thickening. The goodness of fit was 0.9994, indicating a strong correlation between the node thickness increase and the ultimate pull-out resistance increase.

### 4.4. Influence of Different Thicknesses of Strengthened Nodes on Geogrid Strains

**Figure 16:**
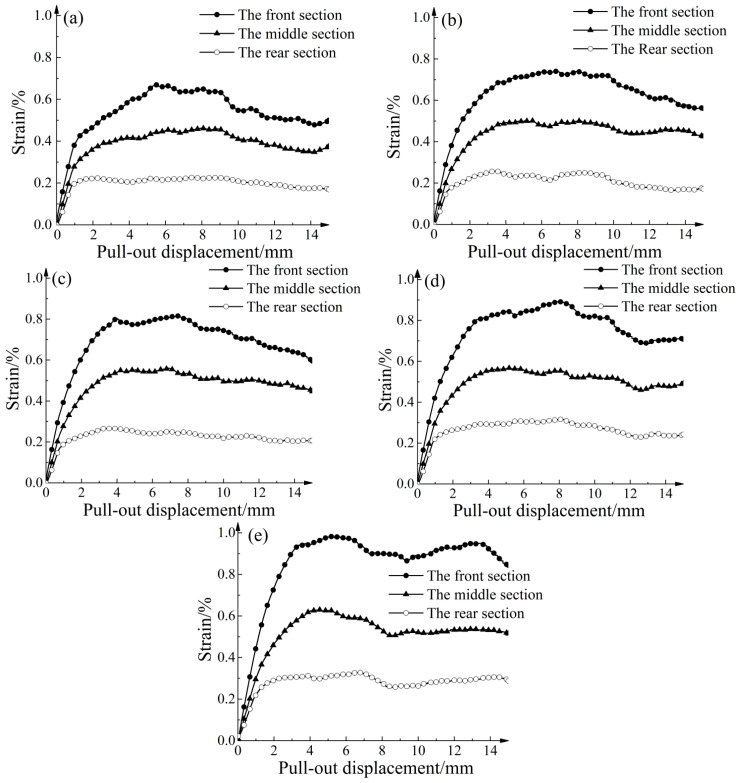
Strain law of geogrids with different node thicknesses. (**a**) Condition A. (**b**) Condition C. (**c**) Condition E. (**d**) Condition F. (**e**) Condition G.

Along the pull-out direction, the strains of the geogrid exhibited significant variation. As shown in Figure 4b, the geogrid was divided into three sections: front, middle, and rear, each with a length of approximately 93.3 mm. Figure 16 illustrates the strain behavior of the geogrid with different strengthened node thicknesses under 30 kPa. Generally, the strain was highest in the front section, followed by the middle section, while the rear section exhibited the lowest strain. In Condition G, the peak strains of the front, middle, and rear sections increased by 46.72%, 37.25%, and 45.78%, respectively. The strengthened nodes significantly increased the strain rates of each section, effectively utilizing the reinforcing performance of the geogrid.

**Figure 17:**
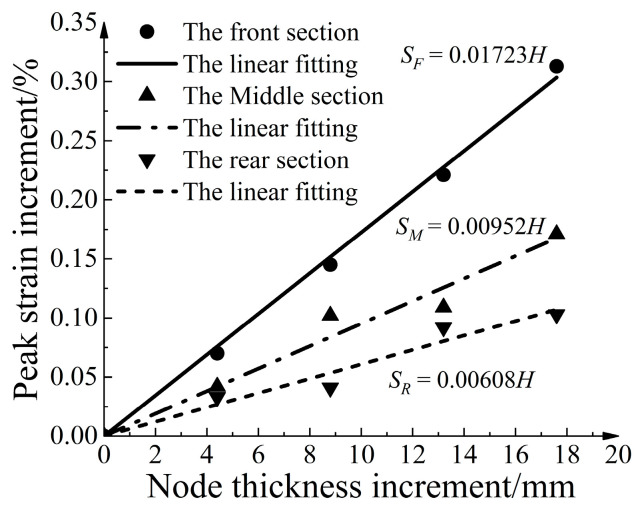
Curve of relationship between peak strain increase and node thickness increase.

Figure 17 illustrates the relationship between the peak strain increase for each section and the strengthened node thickness increase. The relationship between the peak strain increase for each section and the strengthened node thickness increase shows a good linear correlation. The slope of the linear fitted equations indicates that the peak strain increase in the front section was 1.81 times greater than that in the middle section and 2.83 times greater than that in the rear section. This indicates that the influence becomes more significant as the section becomes closer to the pull-out end.

### 4.5. Influence of Different Thicknesses of Strengthened Nodes on Porosity at the Geogrid–Soil Interface

Table 7 presents the changes in local porosity at the geogrid–soil interface and specimen volume before and after the pull-out test. For Condition A, the porosity of the geogrid–soil interface increased by 7.82% after the pull-out, and the volume of the aeolian sand sample increased by 0.28%. In Condition G, the porosity of the interface increased by 9.30%, and the sample volume increased by 0.70%. Compared with the conventional geogrid, the strengthened geogrid exhibited a greater disturbance on the aeolian sand at the interface, resulting in a more pronounced overall shear expansion trend of the sample.

### 4.6. Influence of Different Thicknesses of Strengthened Nodes on Contact Force Chains

**Figure 18:**
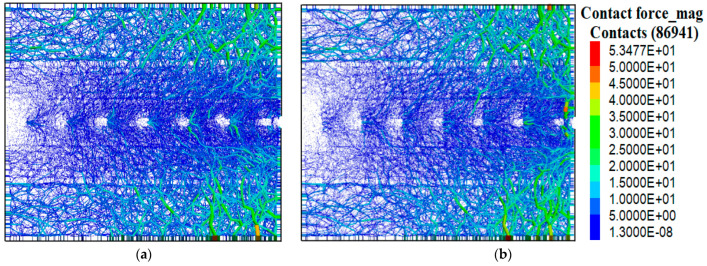
The distribution of force chains under two conditions. (**a**) Condition A. (**b**) Condition G.

Figure 18 illustrates the distribution of contact force chains when the pull-out displacement reached 15 mm. The contact force chains were projected onto the XOZ plane. Overall, the contact network in Conditions A and G exhibits similar distribution patterns. There were noticeable stress voids near the back wall and stress concentration near the front wall. The stress concentration became more pronounced as it approached the front wall. The force chains exhibited a sawtooth distribution near the transverse ribs. Compared with Condition A, Condition G exhibited a more pronounced stress concentration near the pull-out end, indicating that the strengthened-node geogrid had a greater influence on the aeolian sand.

To quantitatively analyze the contact force network, the concept of the probability density function (PDF) of normalized normal contact forces was introduced. The PDF represents the probability of contact forces occurring within a specific range. It can be statistically analyzed through Equation (4), where *dx* = *d*($f_{n}/\bar{f_{n}}$) = 0.1.

(4) $$\left\{\begin{array}{c} \int_{-\infty }^{+\infty }p(x)dx=1\\ p(a<x\leq b)=\int_{a}^{b}p(x)dx \end{array}\right.$$

**Figure 19:**
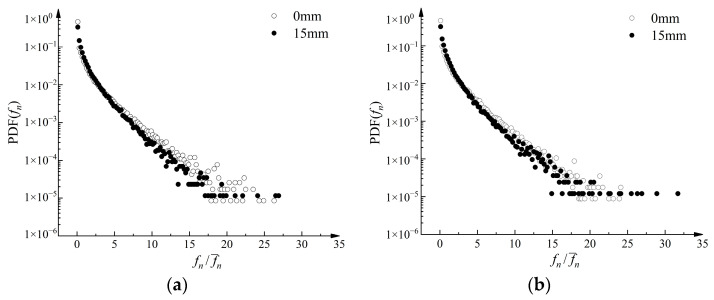
Probability density function curve of normal contact forces. (**a**) PDF in Condition A. (**b**) PDF in Condition G.

In Figure 19a, the probability density of normal contact forces exhibits an exponential distribution. When $f_{n}/\bar{f_{n}}$ exceeded 15, the probability density distribution became irregular. In the range of 0.1 to 3, the probability density after pull-out was higher. This suggests that the average normal contact force after pull-out is greater, leading to an increase in the number of normal contact forces below the average value. Furthermore, Figure 19b demonstrates that the strengthened geogrid exhibited a higher frequency of strong contacts, where the normal contact forces exceeded 25 times the average normal contact force after pull-out.

Figure 19 depicts a clear distribution graph of the contact force network in aeolian sand. However, not all contacts were classified as strong contacts. Typically, the number of weak contacts exceeds that of strong ones, but it is primarily the strong contacts that bear the force transmission within the granular assembly. Therefore, a quantitative analysis of the strong contacts was necessary. Following the study by Otto et al. [28], three criteria were applied to determine the strong contact force chains:

1. The force chain should consist of three or more particles.
2. The absolute value of the major principal stress of each particle in the force chain must be greater than the average value of all particles in the entire model.
3. The angle between adjacent contacts on the force chain should be below a specified threshold *θ_c_*, which is determined by the average coordination number (*Z*) of the model:

*θ_c_* = 180°/*Z* (5)

**Figure 20:**
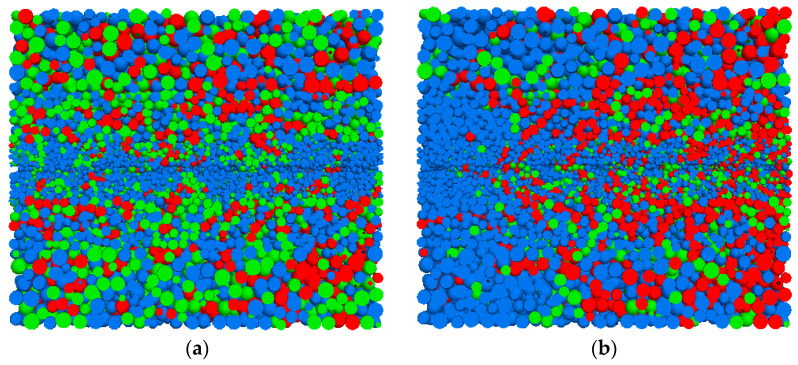
Distribution map of high-stress particles and force chain particles. (**a**) Pulling out 0 mm. (**b**) Pulling out 15 mm.

The particles that satisfy the second criterion are classified as high-stress particles. Based on the three criteria, the internal contacts within the granular assembly are identified. Strong contacts between particles are utilized in a sequential contact search to record contacts that satisfy the criteria, thereby forming complete force chains. Figure 20 illustrates the distribution of high-stress particles (marked in green) and force chains (marked in red) for Condition A. Before pull-out, the distribution of high-stress particles and force chains was relatively uniform. After pull-out, they became more concentrated on one side of the pull-out end. Additionally, there was an increase in the proportion of force chains.

**Figure 21:**
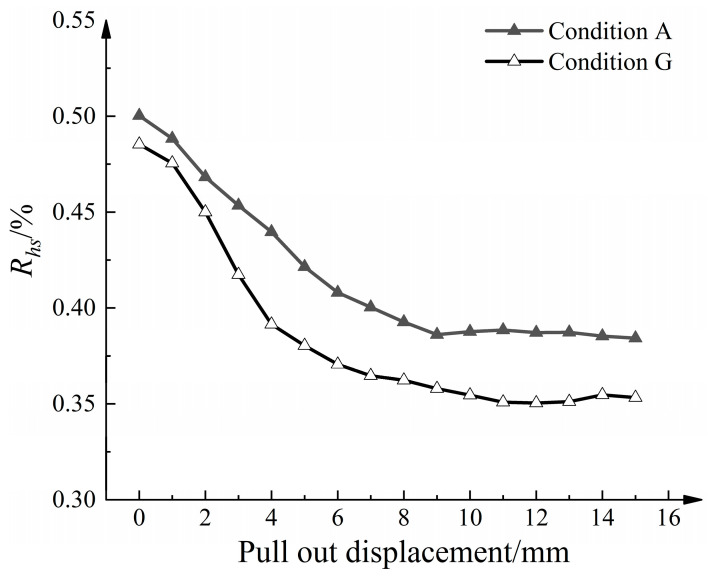
Relationship curve between high-stress particle content and pull-out displacement.

**Figure 22:**
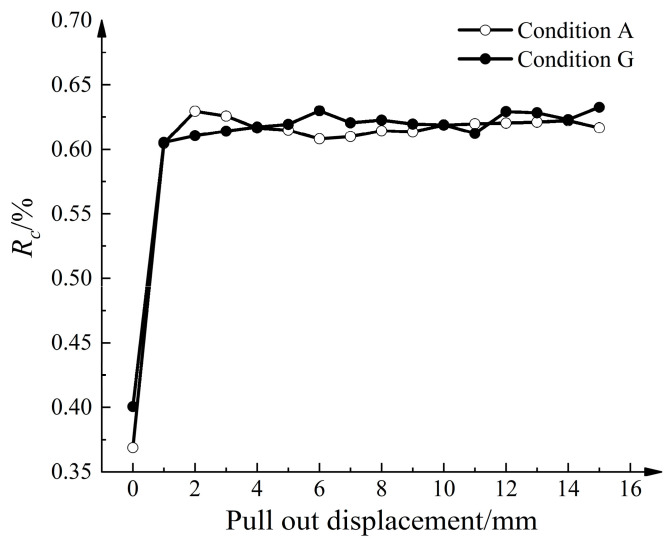
Relationship curve between strong force chain particle content and pull-out displacement.

To investigate the composition of the strong force chain and its development in the model, the aggregate amount ratio was evaluated according to the following equations:

(6) $$R_{hs}=\frac{M_{hs}}{M}$$

(7) $$R_{c}=\frac{M_{c}}{M_{hs}}$$

where *R_hs_* is the percentage of high-stress particle content in the model, *M* is the total mass of aeolian sand, *M_hs_* is the mass of the high-stress particles, *R_c_* is the percentage of the strong force chain content in high-stress particles, and *M_c_* is the mass of the strong force chain content. Figure 21 shows the relationship between *R_hs_* and the pull-out displacement. As the pull-out displacement increases, *R_hs_* gradually decreases and tends to stabilize. This can be attributed to the increased average normal contact force. However, in Condition G, *R_hs_* decreased at a faster rate and remained generally lower than that in Condition A. This indicates that the disturbance caused by the strengthened nodes affects a larger amount of aeolian sand, leading to accelerated rearrangement and dispersion of internal stress transmission paths. Figure 22 shows the relationship between *R_c_* and the pull-out displacement. *R_c_* experiences rapid growth with increasing pull-out displacement and eventually reaches a stable state. Considering the development trend of *R_hs_*, the absolute number of strong force chain particles remains higher than before the pull-out.

**Figure 23:**
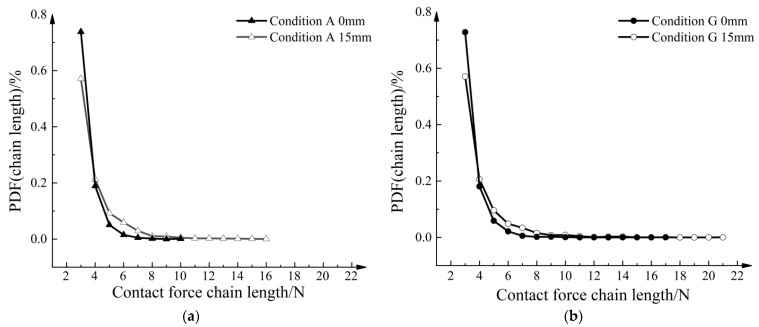
Probability density function curve of contact force chain length. (**a**) Condition A. (**b**) Condition G.

Figure 23a shows the probability density function relationship curve of strong force chain lengths for Condition A. After the pull-out, there is a decrease in the proportion of short force chains composed of three particles, while the proportion of force chains composed of five to seven particles increases. Additionally, long force chains composed of 16 particles emerge. Figure 23b shows the probability density function for Condition G, where long force chains composed of 18 to 21 particles appeared after pull-out. Under the influence of strengthened nodes, longer force chains were formed.

### 4.7. Influence of Different Thicknesses of Strengthened Nodes on Shear Band Range

**Figure 24:**
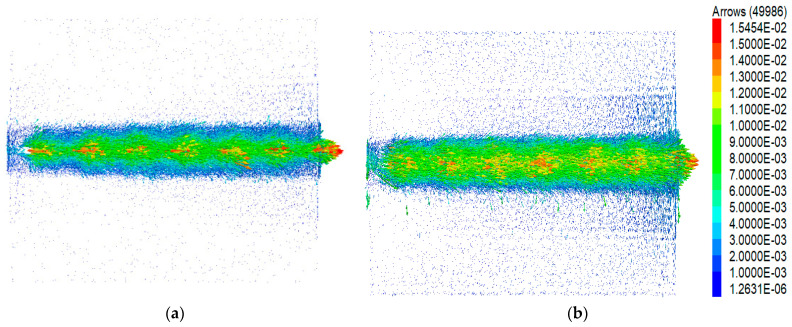
Detail of displacement vectors of aeolian sand particles. (**a**) Condition A. (**b**) Condition G.

Figure 24 illustrates a detailed vector diagram showing the displacements of aeolian sand particles under 30 kPa when pull-out displacement reached 15 mm. Generally, the shear band near the front section exhibited a slightly greater thickness than the shear band near the rear section. Outside the range of the shear band, particle motion was more pronounced near the front section, while particles near the rear section experienced lesser influence. Upon comparing Figure 24a,b, the shear band range in Condition G is larger than that in the conventional geogrid, and the particle motion outside the shear band range is more intense.

**Figure 25:**
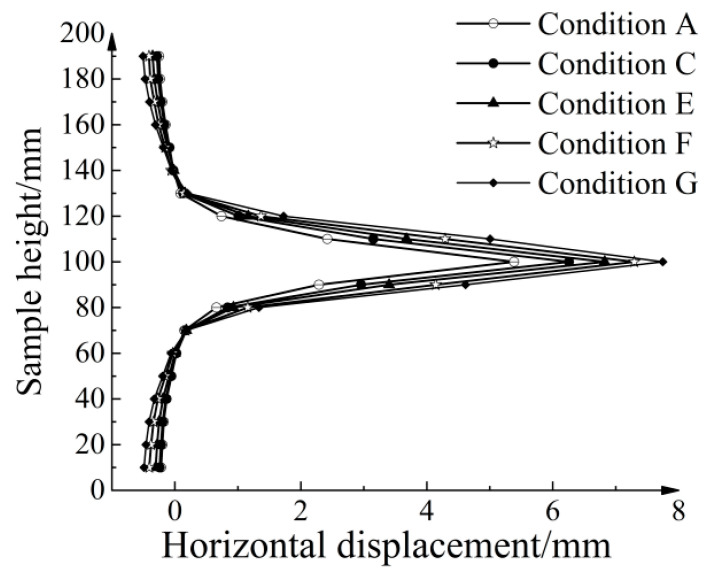
Average horizontal displacement of particles at different heights.

Qualitative analysis of particle displacement provides a preliminary understanding but lacks the ability to evaluate the three-dimensional reinforcement effect of a geogrid under various conditions. To quantitatively assess the influence of different node thicknesses on the shear band range, Figure 25 illustrates the average horizontal displacements of aeolian sand at different height levels. Each height level corresponds to a 10 mm interval. Beyond a certain distance from the geogrid–soil interface, the mean horizontal displacements become negative, indicating a backward movement of most particles at these levels. When pull-out displacement reaches 15 mm, the overall shear band thickness for Conditions A, C, E, F, and G are 36.36, 39.37, 41.35, 44.59, and 47.67 mm, respectively. The boundary for dividing the shear band was set to an average horizontal displacement of 1 mm. Compared with the conventional geogrid, the shear band ranges for the four types of strengthened geogrids expanded to 1.08, 1.14, 1.23, and 1.31 times, respectively. Additionally, the maximum mean horizontal displacements increased to 1.16, 1.27, 1.35, and 1.44 times, respectively. As the node thickness increased, the distribution range of shear bands became larger, and the maximum mean horizontal displacements increased. This indicates that the strengthened nodes not only expand the influence range of shear bands but also enhance the geogrid’s ability to restrict soil movement.

### 4.8. Influence of Different Thicknesses of Strengthened Nodes on Microstructure

To examine the distribution pattern of force chains, the directions of force chains were projected onto the X–Y plane and divided into different sectors based on their angles. By collecting contact information along the interface with a thickness of 50 mm, the number and average force of each sector could be obtained. The black line in Figure 25 represents the average force in each sector. The red dashed line represents the fitted curve using the formula proposed by Rothenburg et al. [29]:

(8) $$F_{n}(\theta )=F_{0}[1+a_{n}\cos2(\theta -\theta_{n})]$$

where $F_{n}(\theta )$ is the average distribution function of contact forces, $F_{0}$ is the average contact force within the statistical range, $a_{n}$ is the anisotropy coefficient, and $\theta_{n}$ is the main direction of anisotropy in the average contact force.

**Figure 26:**
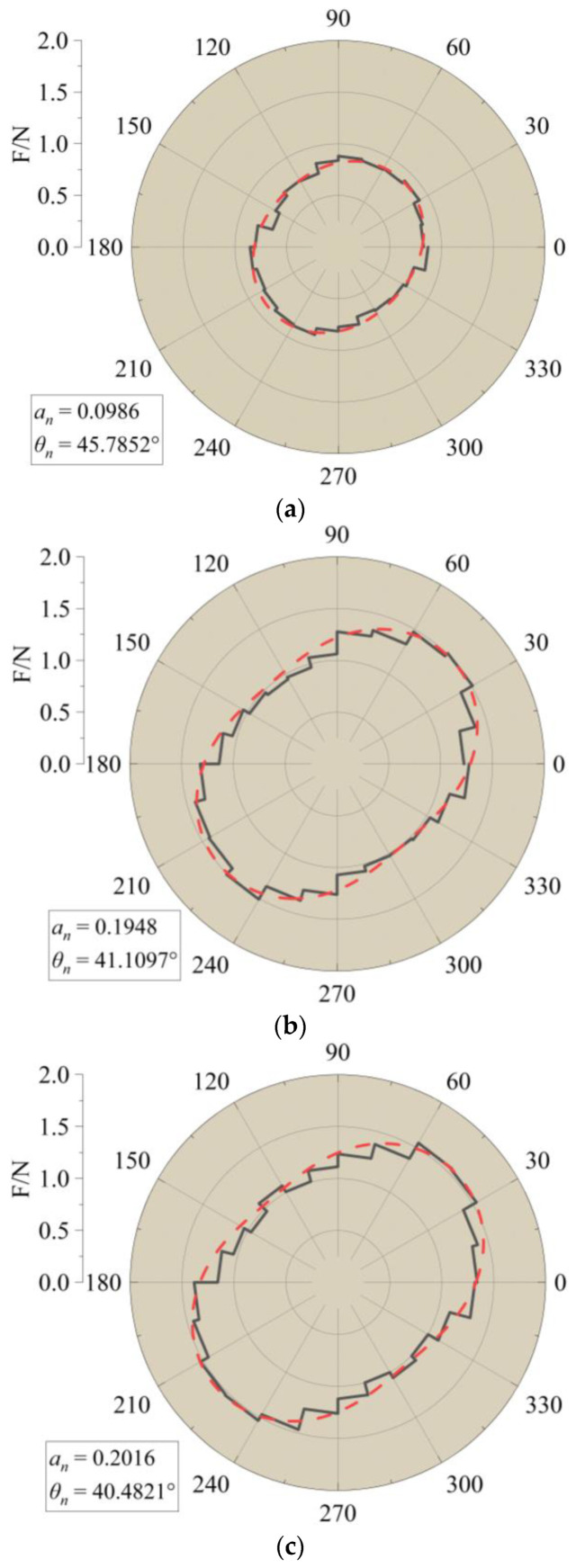

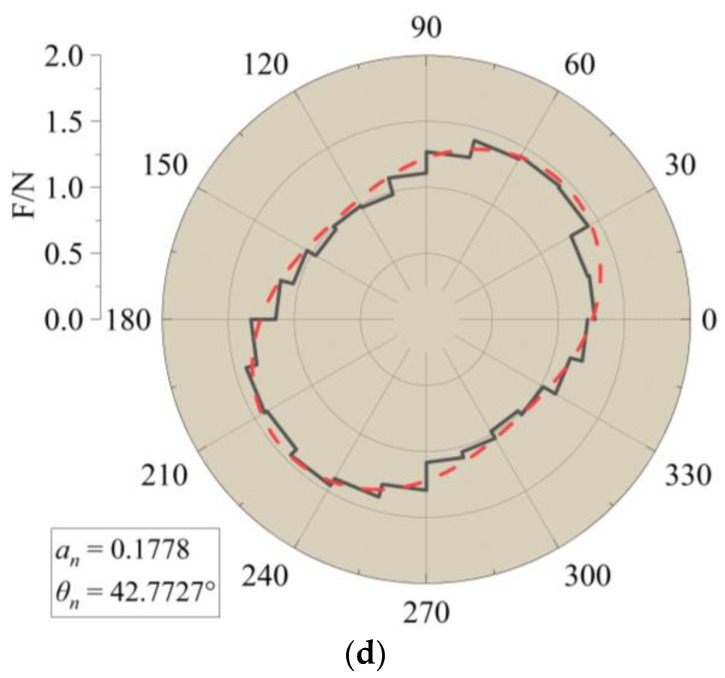
Evolution law of meso fabric in Condition A. (**a**) Before pull-out (0 mm). (**b**) When pull-out displacement reaches 5 mm. (**c**) When pull-out displacement reaches 10 mm. (**d**) When pull-out displacement reaches 15 mm.

**Figure 27:**
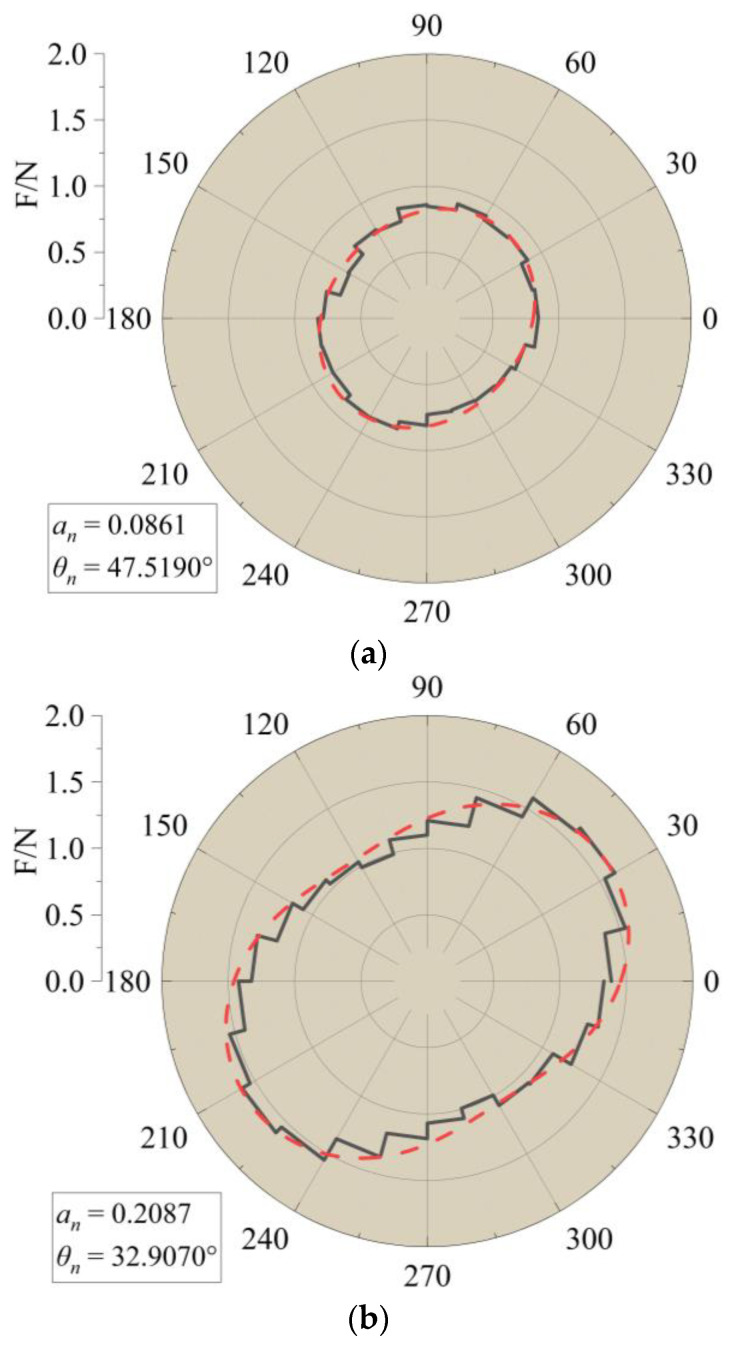

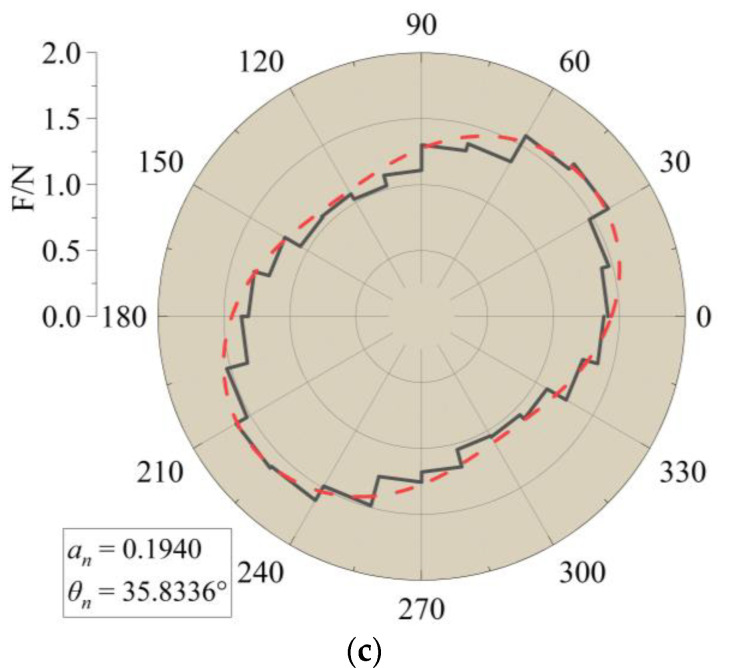

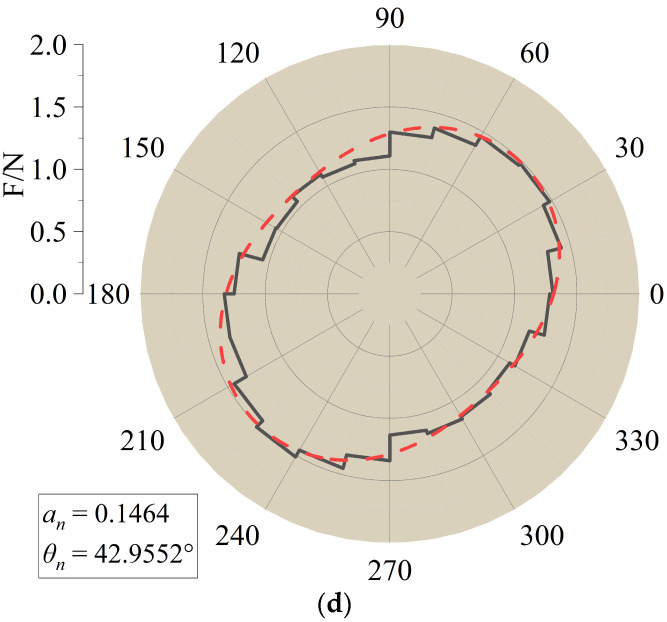
Evolution law of microstructure in Condition G. (**a**) Before pull-out (0 mm). (**b**) When pull-out displacement reaches 5 mm. (**c**) When pull-out displacement reaches 10 mm. (**d**) When pull-out displacement reaches 15 mm.

As the pull-out displacement increased, the principal direction of normal contact force of aeolian sand particles demonstrated an initial clockwise deviation followed by a counterclockwise deviation. The anisotropy coefficients exhibited a pattern of initially increasing and then decreasing, indicating a negative correlation with the principal direction. In Figure 26 and Figure 27, the microstructural development trend among different conditions is similar, but there are also some differences. For instance, the minimum principal direction of contact force in Condition G reached 32.9°; in Condition A, it reached a minimum of 40.5°. When the pull-out displacement reached 5 mm, the anisotropy coefficients were still increasing in Condition A; in Condition G, they already started to decrease.

### 4.9. Influence of Strengthened Node Quantity and Distribution Patterns on Pull-out Resistance

**Figure 28:**
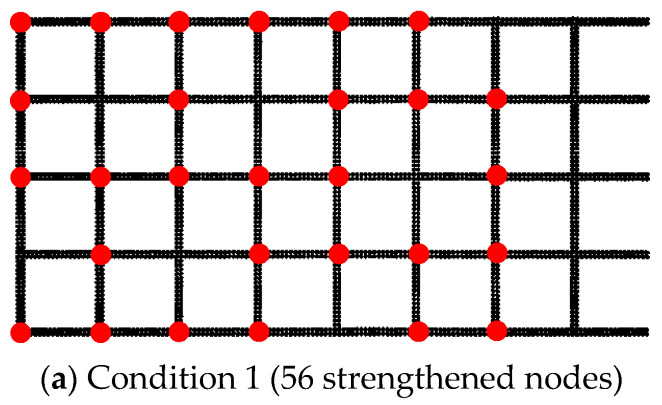

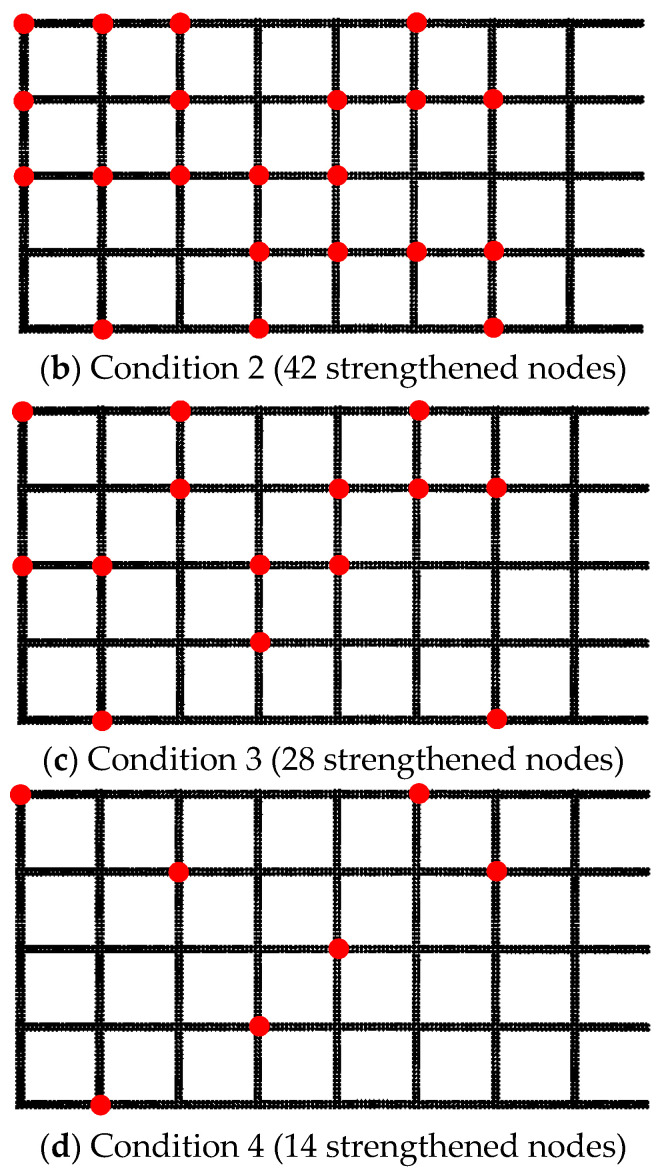
Random distribution of different numbers of strengthened nodes.

To examine the correlation between the number of strengthened nodes and the ultimate pull-out resistance increase under 30 kPa, Condition C served as the baseline. By randomly removing strengthened nodes, four conditions were obtained, as depicted in Figure 28. The red circles in the figure represent the strengthened nodes.

**Figure 29:**
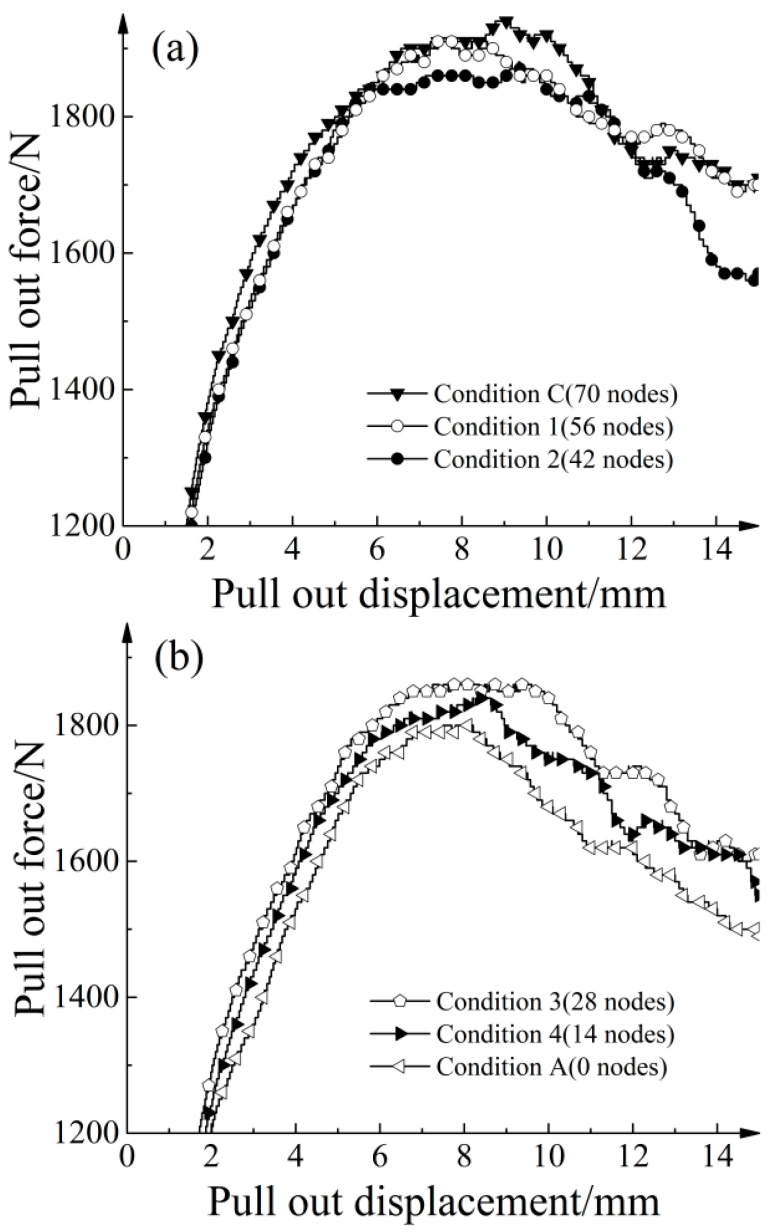
Pull-out results with different numbers of strengthened nodes under random distribution.

Figure 29 presents the pull-out force versus displacement curves for different quantities of randomly distributed strengthened nodes. The results of Figure 29 were obtained by testing various working conditions in Figure 28. A larger amount of strengthened nodes resulted in a steeper slope in the initial stage of the curve and a greater increase in the ultimate pull-out resistance. All seven transverse ribs were positioned within the effective zone, as depicted in Figure 4b. By maintaining one single row of strengthened nodes and assuming that the strengthened nodes on the transverse rib do not exert influence on one another, each node could be considered to apply independently in this regular distribution. Each row consisted of 10 strengthened nodes. When only one single row of strengthened nodes was maintained, multiple pull-out tests were simulated under 30 kPa. Table 8 presents the corresponding results. The minimum increase in ultimate pull-out resistance provided by the strengthened nodes was 31.6 N, while the maximum increase was 60.9 N. The different quantities of strengthened nodes at different spatial locations resulted in varying ultimate pull-out resistance increases, indicating a no-uniform distribution of shear stresses along the geogrid–soil interface.

### 4.10. Discussion on the Collective Effect of Strengthened Nodes

**Figure 30:**
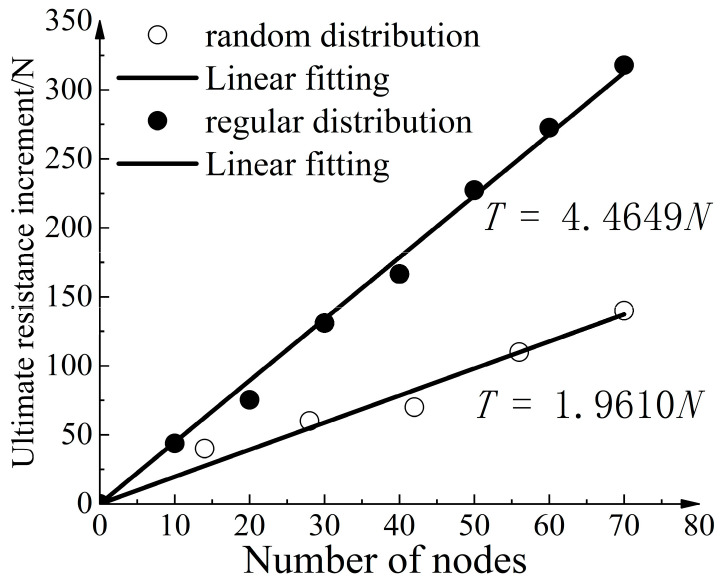
Relationship curve between number of strengthened nodes and ultimate pull-out resistance increase.

Figure 29 depicts the relationship between the number of strengthened nodes and the ultimate pull-out resistance increase for different distribution patterns. Under a random distribution, the quantity of strengthened nodes (*N*) exhibits a linear relationship with the ultimate pull-out resistance increase (*T*). The relationship can be represented by the following linear fitting equation: T=1.9610N. The goodness of fit was 0.9917, indicating a strong linear correlation between the two variables. Utilizing the data from Table 8, the ultimate pull-out resistance increases provided by each row of strengthened nodes were summed. The relationship between the quantity of strengthened nodes and the ultimate pull-out resistance increase under regular distribution was obtained. From Figure 30, the curve could be linearly fitted as T=4.4649N, with a goodness of fit of 0.9985. Notably, for the same number of strengthened nodes, the cumulative increases of ultimate pull-out resistance under regular distribution were greater than that under random distribution. This indicates the presence of a collective effect when multiple nodes simultaneously apply, which reduces the rate of ultimate pull-out resistance increase per unit quantity of strengthened nodes.

**Figure 31:**
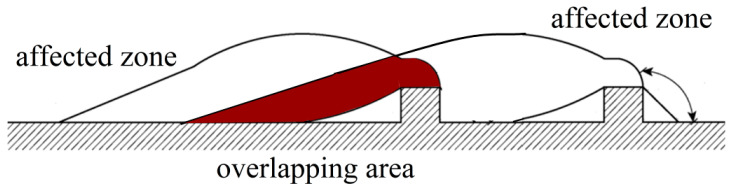
Analysis of slip fracture surface of two strengthened nodes.

As depicted in Figure 31, the influence zones of two adjacent nodes on the longitudinal rib overlapped with each other. Therefore, the combined influence zone was smaller than the sum of the individual influence zones, resulting in a collective effect. The collective effect of strengthened nodes contributed to a decrease in the ultimate pull-out resistance. The collective effect explains the results shown in Figure 11. When the material is consistent, strengthening on both sides simultaneously, as opposed to strengthening nodes on one side, decreases the proportion of the overlapping area within the influence zone, thereby diminishing the collective effect. Consequently, it becomes the optimal method among the three node-thickening methods.

**Figure 32:**
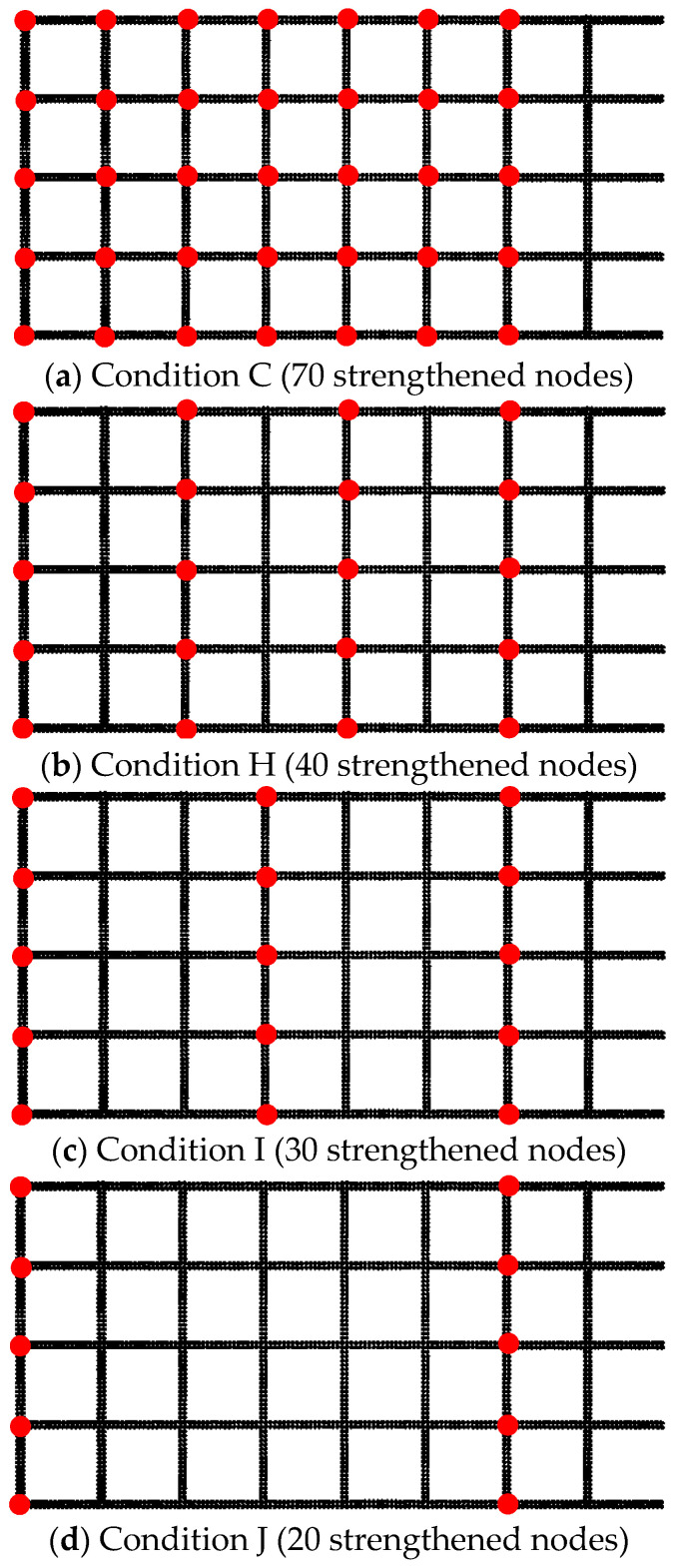
Design conditions of different strengthened node spacings.

When the spacing between two adjacent strengthened nodes is too small, the influence zones significantly overlap, leading to a significant collective effect. If the spacing between nodes is moderate, adjacent nodes independently apply without exerting influence on each other, thereby avoiding the influence of collective effects. To study the influence of strengthened node spacings on the collective effect, Figure 32 shows four design conditions. The collective effect reduction coefficient serves as a quantitative indicator to evaluate the strength of the collective effect. It can be calculated using the following formula:

(9) $$\eta =\frac{t_{s}}{t_{r}}$$

**Figure 33:**
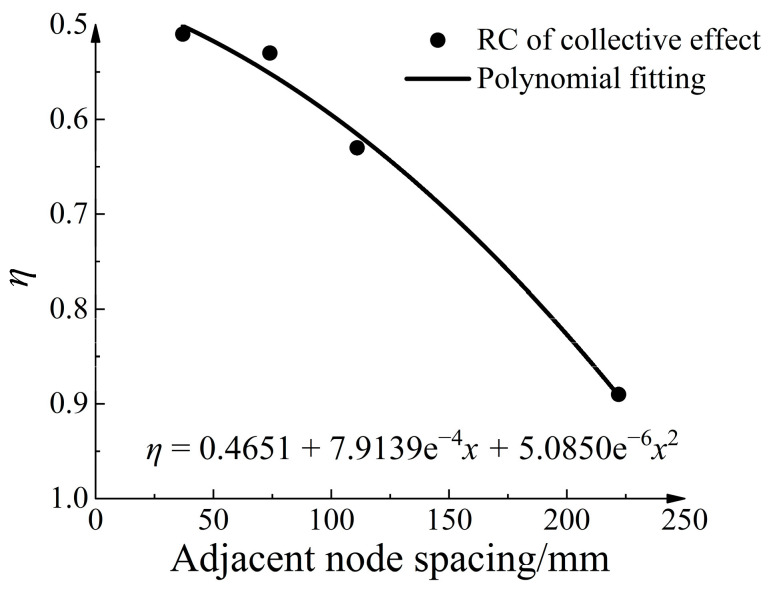
Relationship between collective effect reduction coefficient and adjacent strengthened node spacing.

Here, *t_r_* represents the ultimate pull-out resistance increase when the strengthened node separately exists, and *t_s_* represents the average increase of ultimate pull-out resistance when nodes are randomly distributed. Table 9 shows the collective effect reduction coefficients under different conditions. Additionally, Figure 33 shows the relationship between the collective effect reduction coefficient and the spacing between adjacent nodes. As the spacing between adjacent nodes increases, the overlap of the influence zones of adjacent strengthened nodes decreases, leading to a gradual decrease in the collective effect reduction coefficient. A polynomial fitting was performed on the collective effect reduction coefficient, and the fitting equation was as follows:

(10) $$\eta =0.4651+7.9139e^{-4}x+5.0850e^{-6}x^{2}$$

The goodness of fit was 0.975. Based on the fitting equation, the optimal node spacing could be predicted. When η = 1, the adjacent node spacing was calculated to be 255 mm. This means that when the spacing between adjacent nodes exceeds 255 mm, the increase in ultimate pull-out resistance provided by multiple nodes does not reduce.

## 5. Conclusions

Based on the results of triaxial tests of aeolian sand, tensile tests of a single geogrid, and pull-out tests of a geogrid in aeolian sand, a discrete element numerical model was developed for pull-out tests. The macroscopic and microscopic characteristics of the geogrid–soil interface were investigated, leading to the following conclusions:

(1) Compared with the homogeneous aeolian sand numerical model, the layered aeolian sand numerical model showed closer agreement with the experiment results, confirming the rationality and superiority of the layered aeolian sand numerical model. Based on the insertion shear theory, the calculated values of the ultimate pull-out resistance increase provided by the strengthened nodes were close to the numerical solution, demonstrating the rationality of the node simulation method.
(2) When multiple strengthened nodes are simultaneously applied, if the spacing between adjacent strengthened nodes is small, the influence zones of the nodes overlap, leading to a collective effect. As the spacing between adjacent strengthened nodes increases, the collective effect of the nodes gradually diminishes.
(3) Different node-thickening methods affect the reinforcement performance of the geogrid. When the node thickness remains constant, strengthening on both sides reduces the proportion of the overlapping area in the influence zone, thereby weakening the collective effect.
(4) When the node thickening method is consistent, increasing node thickness leads to a corresponding increase in the pull-out resistance increase provided by the strengthened nodes. There exists a linear relationship between the ultimate pull-out resistance increase and the node thickness increase.
(5) The peak strain increases of the front, middle, and rear sections of the geogrid exhibited a good linear relationship with the node thickness increase. The growth rate of the peak strain in the front section was 1.81 times higher than that in the middle section, and it was 2.83 times higher than that in the rear section.
(6) Condition G, which utilized geogrids with strengthened nodes, showed a 1.31 times wider shear band range compared with conventional geogrids, demonstrating a significant three-dimensional reinforcement effect. Additionally, the maximum mean horizontal displacement increased by 1.44 times, indicating a greater capacity to restrain soil mass displacement.

## Figures and Tables

**Table 1:** Physical parameters of aeolian sand.

Parameter	Value	Parameter	Value
Specific gravity	2.70	Maximum dry density (g·cm^−3^)	1.71
Unit weight (kN·m^−3^)	15.8	Maximum void ratio	0.92
Natural moisture content (%)	0.9	Minimum void ratio	0.54
Optimum moisture content (%)	14.0	Cohesion (kPa)	0
Density (g·cm^−3^)	1.58	Internal friction angle (°)	27.4

**Table 2:** Mesoscopic parameters of flexible films.

Parameter	Value
Deformation modulus (Pa)	1 × 10^6^
Normal contact stiffness *kn* (N·m^−1^)	3927
Tangential contact stiffness *ks* (N·m^−1^)	2617
Bond strength (N·m^−2^)	1 × 10^300^
Membrane particle radius (mm)	2.5

**Table 3:** Calibration parameters of aeolian sand.

Parameter	Value	Parameter	Value
Deformation modulus (MPa)	80	Friction coefficient	0.55
Stiffness ratio	5.0	Rotational resistance coefficient	0.15
Radius coefficient	1.0	Porosity	0.40

**Table 4:** Macro parameters of geogrid.

Aperture size (mm)	34 × 31	Transverse rib width (mm)	5.00
Longitudinal rib width (mm)	3.00	Transverse rib thickness (mm)	1.60
2% elongation tensile strength (kN·m^−1^)	21.20	Ultimate tensile Strength (kN·m^−1^)	46.92
5% elongation tensile strength (kN·m^−1^)	37.96	Longitudinal rib thickness (mm)	3.80

**Table 5:** Meso parameters of geogrid.

Density (kg·m^−3^)	1000	Damping ratio	0.7
Deformation modulus (MPa)	3.3 × 10^3^	Friction coefficient	0.5
Particle size of transverse ribs (mm)	1.97	Normal bonding strength (N·m^−2^)	4.2 × 10^10^
Particle size of longitudinal ribs (mm)	2.03	Tangential bonding strength (N·m^−2^)	4.2 × 10^10^
Particle size of nodes (mm)	2.2	Radius coefficient	1.0

**Table 6:** Interface strength parameters under different node -thickening methods.

Method	Linear Fitting Expression	Quasi Cohesion (kPa)	Interface Friction Angle (°)
Condition B	$\tau_{f}=0.7020f^{*}+5.0340$	5.0340	35.0688
Condition C	$\tau_{f}=0.7214f^{*}+5.1630$	5.1630	35.8067
Condition D	$\tau_{f}=0.6898f^{*}+5.6035$	5.6035	34.5979
Condition A	$\tau_{f}=0.6404f^{*}+5.2965$	5.2965	32.6355

**Table 7:** Local porosity and sample volume change at the interface between reinforcement and soil.

Condition	Porosity before Pulling	Porosity after Pulling	Sample Height before Pulling (mm)	Specimen Height after Pulling (mm)
Condition A	0.4563	0.4920	214.580	215.176
Condition C	0.4541	0.4954	214.342	215.380
Condition E	0.4523	0.4977	214.374	215.612
Condition F	0.4556	0.4981	214.594	215.820
Condition G	0.4549	0.4972	214.596	216.088

**Table 8:** Ultimate pull-out resistance increase of strengthened nodes under regular distribution.

Position	Row 1	Row 2	Row 3	Row 4	Row 5	Row 6	Row 7
Distance from front wall (mm)	27	64	101	138	175	212	249
Ultimate pull-out force increase (N)	45.4	45.1	60.9	35.5	55.6	31.6	43.8

**Table 9:** Effect of different strengthened node spacings on collective effect.

Different Working Conditions	Cond C	Cond H	Cond I	Cond J
Adjacent strengthened node spacing (mm)	37	74	111	222
Ultimate pull-out resistance (N)	1960	1894	1885	1879
Ultimate pull-out resistance increase (N)	160	94	85	79
Increase when the node exist separately (N)	313	179	134	89
Reduction coefficient of collective effect	0.51	0.53	0.63	0.89

## Data Availability

Not applicable.
